# Development of a High-Efficient Mutation Resource with Phenotypic Variation in Hexaploid Winter Wheat and Identification of Novel Alleles in the *TaAGP.L-B1* Gene

**DOI:** 10.3389/fpls.2017.01404

**Published:** 2017-08-10

**Authors:** Huijun Guo, Zhihui Yan, Xiao Li, Yongdun Xie, Hongchun Xiong, Yunchuan Liu, Linshu Zhao, Jiayu Gu, Shirong Zhao, Luxiang Liu

**Affiliations:** National Engineering Laboratory of Crop Molecular Breeding and National Center of Space Mutagenesis for Crop Improvement, Institute of Crop Sciences, Chinese Academy of Agricultural Sciences Beijing, China

**Keywords:** wheat, phenotypic mutation, novel allele, gene *TaAGP.L-B1*, starch content, genetic resource

## Abstract

Mutated genetic resources play an important role in gene/allele characterization. Currently, there are few hexaploid winter wheat mutated resources available. Here, we developed a hexaploid winter wheat resource by inducing mutations via EMS treatment by the single seed descent method. A broad mutation spectrum with high mutation frequency (∼19%) on phenotypic variations was identified. These mutations included spike, leaf and seed morphology, plant architecture, and heading date variations. To evaluate the efficiency of the resource for reverse genetic analysis, allelic variations in the *TaAGP.L-B1* gene, encoding the AGPase large subunit, were screened by the TILLING approach. Four missense mutations were identified and one allele in line E3-1-3, resulted in an amino acid change predicated to have severe effects on gene function. The other three mutations were predicted to have no effect. Results of gene expression patterns and grain starch content demonstrated that the novel allele in E3-1-3 altered the function of *TaAGP.L-B1*. Our results indicated that this mutated genetic wheat resource contained broad spectrum phenotypic and genotypic variations, that may be useful for wheat improvement, gene discovery, and functional genomics.

## Introduction

Genetic resources played a tremendous role in the first green revolution, providing dwarfing, disease resistance, and yield-improving genes (Hoisington et al., 1999). Forward and reverse genetic approaches have identified novel genes and gene functions that have proved important for new or improved genetic-based plant traits. An example of this is the highly resistant starch mutant in rice created for improving human health that utilized map-based cloning to discover the function of the soluble starch synthase gene, *SSIIIa*, in combination with the granule-bound starch synthase *Waxy^a^* allele to play a critical role in resistant starch synthesis (Zhou et al., 2016). A few other examples include the shrunken rice mutant that demonstrates how the *OsAGPL2* gene plays an essential role for storage substance accumulation (Tang et al., 2016) and the wheat mutant gene *MNR220* that carries a broad-spectrum disease resistance locus that enhances resistance to powdery mildew and three rust species (Campbell et al., 2012). Numerous SNPs and novel alleles have been targeted for gene characterization using functional genomics tools in model plants putatively useful for crop improvements (Hadrich et al., 2011; Hazard et al., 2012).

Many mutagenized populations have been created in several species such as soybean (Tsuda et al., 2015), rice (Wu et al., 2005; Till et al., 2007; Suzuki et al., 2008; Serrat et al., 2014), maize (Till et al., 2004), and sorghum (Xin et al., 2008). These resources have been used for novel gene/allele mining and morphological variation screening. Wheat is classified into diploid, tetraploid, and hexaploid wheat and further split into spring and winter wheat according to ploidy and growth habits. It has genomic redundancy and a large genome size. Due to these characteristics, a single gene mutation would not likely result in phenotypic changes. Numerous mutant populations would need to be developed using multi-polymorphic wild types to account for this redundancy and present changes in phenotypes. Recently, mutated wheat populations have been developed from several genotypes. Under a tetraploid background, *Kronos* (Slade et al., 2005; Uauy et al., 2009), *Marco Aurelio* (Colasuonno et al., 2016), and *Svevo* (Bovina et al., 2014) have been used as the wild type varieties to develop the mutagenized population. For hexaploid spring wheat, seven genotypes from four countries have been used as the background genotype. These include *Cadenza* (Rakszegi et al., 2010) and a breeding line “*UC1041*+*Gpc-B1/Yr36*” (Uauy et al., 2009) from the United Kingdom, *Chara* (Fitzgerald et al., 2015), *QAL2000* (Dong et al., 2009) and *Ventura* (Dong et al., 2009) from Australia, and *Express* (Slade et al., 2005) and *Indian* (Dhaliwal et al., 2015) from the United States. In hexaploid winter wheat, only one mutated population based on cultivar *Jinmai 47* (drought tolerant) has been created (Chen et al., 2012). Allelic mutations have been identified in these populations, while phenotypic variations only are screened in populations from *Svevo*, *Cadenza*, *Indian*, and *Jinmai 47*. Additional mutated populations from polymorphic genetic backgrounds and various environmental growth conditions should be used in reverse genetic research to build upon our knowledge of wheat genetic plasticity. An ideal candidate for mutated germplasm is hexaploid winter wheat as it is grown and consumed globally. Greater understanding of wheat morphology variation could lead to innovative mutant germplasm resources to ensure crop preservation.

Starch is the major component of wheat endosperm and has a large impact on grain yield and quality characteristics. Its synthesis and accumulation is an intricate biological process that is regulated by multiple enzymes. Adenosine diphosphate glucose phosphorylase (AGPase, EC: 2.7.7.27) is one of these enzymes and catalyzes the conversion of glucose-1-phosphate (G-1-P) to ADP-glucose (ADP-Glc) (Tuncel and Okita, 2013). ADP-Glc is continuously converted to starch by a series of enzymes including soluble starch synthase and starch branching enzymes (Keeling and Myers, 2010). In wheat, AGPase consists of two large and two small subunits (Danishuddin et al., 2011; Saripalli and Gupta, 2015) and overexpression of the large subunit gene not only increases starch biosynthesis in the endosperm of wheat (Kang et al., 2013), but also stimulates photosynthesis and carbon metabolism (Smidansky et al., 2007). However, the biological mechanism of the AGPase gene on plant growth and crop productivity is still not very clear (Tuncel and Okita, 2013; Saripalli and Gupta, 2015). Gene mining techniques involving the search for novel alleles or functionality research involving gene mutations would benefit our understanding of this genes role in crop production. The AGPase large subunit gene SNPs and in-dels previously identified are mostly synonymous substitutions or located in non-coding regions. Four SNPs were specifically found to affect seed number and thousand grain weight (TGW), but their effects on starch content were not elucidated (Rose et al., 2016). Even a single missense mutation identified in a mutagenized wheat population might be able to create allelic variation in the resulting AGPase-mutant phenotype (Li S. et al., 2017; Li W. et al., 2017). To better understand its function in starch biosynthesis, yield production and plant development, it is necessary to mine for additional non-lethal alleles.

A non-transgenic, reverse genetic approach referred to as TILLING (Targeting Induced Local Lesions IN Genomes) has been used for mutation discovery and gene function characterization in many plant species in recent years (Till et al., 2003; Slade et al., 2005; Xin et al., 2008; Kim and Tai, 2014). By using this platform, a series of novel alleles have been identified from mutated populations. Genetic effects derived from functional alleles in the gene *GPC-1* (Avni et al., 2014), *SBEII* (Hazard et al., 2012), and *VRN* (Chen and Jorge, 2012) have been analyzed in wheat to enhance the understanding of their biological roles in metabolic networks and their function on agronomic traits.

The present study developed a mutagenized genetic population resource that possessed phenotypic variation using the high-yield winter wheat cultivar *Jing 411* as the wild type. Novel allele in the large subunit gene of AGPase was identified using the TILLING approach in which the missense mutation resulted in reduced starch content in wheat grain.

## Materials and Methods

### Materials

Winter wheat (*Triticum aestivum* L.) cultivar *Jing411* was bred for high yield potential, cold tolerance, high tilling ability, and is frequently planted in the Northern winter wheat region in China. It has become the elite parent in wheat high-yield breeding processes. Seeds of *Jing411* were used for ethyl methane sulphonate (EMS) treatment to induce random mutations.

### EMS Treatment

Dry seeds (**Supplementary Figure S1a**) of *Jing411* were initially soaked in tap water for 10 h until germinated and embryo buds began to emerge out of the seed coat (**Supplementary Figure S1b**). The germinated seeds were then incubated in 0.5, 1.0, or 1.5% EMS in 0.1 M sodium phosphate buffer (pH 7.0) for 4 h. Around 2000 seeds were used in each treatment. EMS treatment was performed on a shaker at 50 rpm in dark conditions at 20°C with one seed per milliliter EMS buffer. After incubation, seeds were washed in tap water for 4 h at room temperature and prepared for sowing.

### Plant Growth Conditions and Harvest

Treated seeds were directly sowed with 5 cm spacing and grown into M_1_ plants. All spikes were bagged to be used strictly in self-crosses. One single M_2_ seed was harvested from each plant. All harvested M_2_ seeds were sowed and grown into M_2_ individual plants. Twenty M_2_ seeds per row were planted in 19 rows with one row wild type *Jing411* wheat. Each M_2_ plant was harvested to develop the M_3_ generation. Twenty M_3_ seeds from individual M_2_ plant were planted into one row and used to verify their phenotype variation.

Row length was 2 m long with 30 cm row-to-row spacing. All plants were grown in the experimental field, Institute of Crop Science, Chinese Academy of Agricultural Sciences, Beijing.

### Screening and Identification of Phenotypic Mutation

Mutations in growth habit, leaf morphology, heading date, and spike morphology were investigated in the M_2_ generation. Heading date ±2 days compared to the wild type was considered a mutant. The plant height, tiller number, spike length, and TGW of all individuals were measured after harvesting and a mean value greater or less than two standard deviations of the wild type mean was classified as a mutant. The mutated phenotype was identified in the M_3_ generation plants.

Mutation frequency was calculated as percentage of total number of mutants divided by corresponding population size.

### Construction of DNA Pool

Young leaves at seedling stage from M_2_ individual plants were collected and genomic DNA was extracted using the DNA-quick Plant System kit (Tiangen Biotech, Beijing, China). The DNA concentration was normalized to 50 ng/μl according to Guo et al. (2017). The DNA was 2×-pooled for gene TILLING according to Till et al. (2006).

### Targeting Induced Local Lesions IN Genomes

Point mutations of the target gene *TaAGP.L-B1* (7725 bp; GenBank accession no: AJ563452) were screened using the TILLING approach following the protocol of Till et al. (2006). Specific primers were designed and used for PCR amplification of this region. Their sequences are: AGP-1F:ATCTGGATACTTGTACATCTGC, AGP-1R: GAAGACATAGACTCCCATAG; AGP-2F: CATTGCAGCCCCCAAAGTTC, AGP-2R: AAAGCGGCGTGGTTTCAGAC. Gene structure and location of the primers were shown in **Supplementary Figure S2**, lengths of the amplicons were 670 and 728 bp respectively, including exons and introns region. Potential mutations were further sequenced to verify nucleotide variation.

Each point mutation was analyzed online by the PARSESNP (Project Aligned Related Sequences and Evaluate SNPs^1^) and SIFT (Sorting Intolerant from Tolerant^2^) programs. Mutations with PSSM > 10 or SIFT < 0.05 are predicted to have a severe effect in protein function (Ng and Henikoff, 2003; Taylor and Greene, 2003).

### Real Time-Quantitative Polymerase Chain Reaction

At 12, 18, and 24 days after flowering (DAF), seeds were sampled for gene expression analysis. Total RNA was extracted using Trizol (Invitrogen, Carlsbad, CA, United States), and cDNA was synthesized with 5×All In One MasterMix kit (With gDNase, ABM, Co.). Primers for RT-qPCR were designed using software Beacon Designer 7.5 (PREMIER, Palo Alto, CA, United States). The following primers were used: AGP-qF: GAACTACATGACTTTGGGTCTGAGA, AGP-qR: CATCATCATCGCATTCTTGAGCT. Amplification was performed with 0.5 μmol primer using SsoFast EvaGreen Supermix kit (Bio-Rad) on a CFX96 system. The program started from an initial denaturation at 95°C for 3 min, and followed by 40 cycles at 94°C for 30 s, annealing for 30 s, and 72°C for 10 s, and a melt curve stage. The *actin* gene (GenBank accession no: AAW78915) was used as an internal control. Three biological and technical replicates were used for each genotype. The relative expression level was calculated using ΔΔ*C*t method (Livak and Schmittgen, 2001). Data were analyzed using a one-way ANOVA with *P* < 0.05 or 0.01 considered as significant.

### Determination of Starch Content

Mature seeds were milled using a cyclone mill (CT410, FOSS Analytical, Co., Ltd) with a 0.5 mm flour screen. Starch content was measured using a Total Starch Assay Procedure (amyloglucosidase/α-amylase method, K-TSTA) from Megazyme (Megazyme International, Ltd, Wicklow, Ireland). Data were analyzed by one-way ANOVA and *P* < 0.05 or 0.01 was considered significant.

## Results

### Broad Mutation Spectrum on Visible Phenotype

**FIGURE 1:**
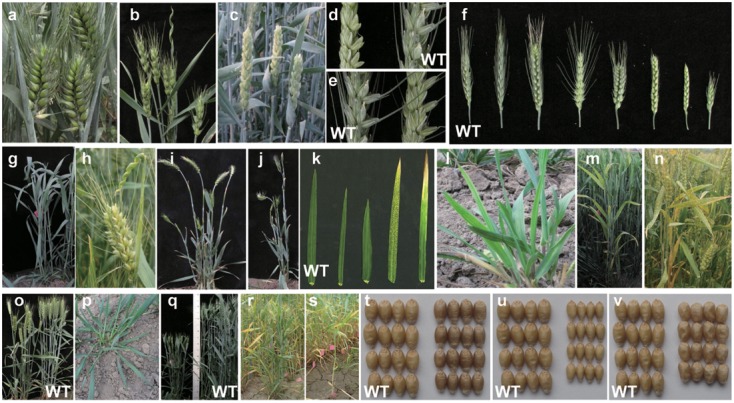
Phenotypic mutations in the mutagenized wheat population. **(a–f)** Represent spike morphology mutations observed in mutagenized wheat. **(a)** Compact spike; **(b)** degenerated spike; **(c)** awnless spike; **(d)** male sterile spike without awn; **(e)** male sterile spike with awn; **(f)** totality of spike mutation phenotypes. **(g–n)** Represent mutation in leaf morphology. **(g,h)** Curly flag leaf; **(i,j)** degenerated leaf; **(k)** leaf size and chlorophyll content variation; **(l)** yellow-striped leaf; **(m)** chlorotic leaf; **(n)** necrotic leaf. **(o–s)** Represent plant architecture mutations observed. **(o)** Waxless plant; **(p)** prostrate growth habit; **(q)** dwarfing plant; **(r)** increased tiller numbers; **(s)** single tiller. **(t–v)** Represent mutations in seed morphology. **(t)** Red grain; **(u)** small grain; **(v)** wrinkle grain.

**FIGURE 2:**
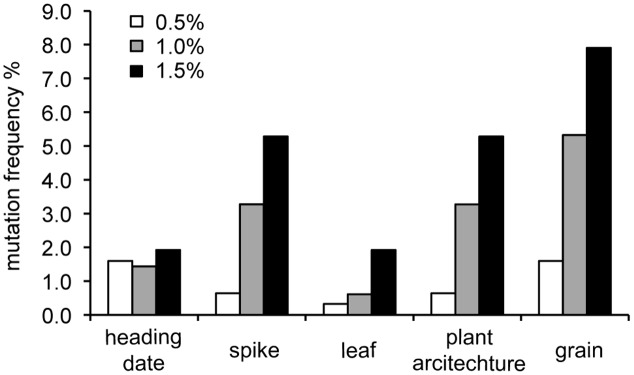
Mutation frequency of each morphological variation category in the three mutagenized wheat populations. Mutations were observed in the heading date, spike, plant architecture, and leaf and grain morphology. Wheat was treated with 0.5% (*n* = 313), 1.0% (*n* = 489), and 1.5% (*n* = 417) EMS to induce mutations. Mutation frequency increased with increased EMS application.

A total of 313, 489, and 417 individual M_2_ plants and their M_3_ lines were investigated at 0.5, 1.0, and 1.5% EMS treatment, respectively. Mutations were observed in the heading date, spike, plant architecture, and leaf and grain morphology. Phenotypic variations in the mutagenized wheat population can be observed in **Figure 1**. The mutation frequency of each morphological variation category grew as EMS concentration increased (**Figure 2** and Supplementary Table S1).

#### Spike Morphology

Mutations on spike morphology manifested in spikelet density, awn character, sterility, and spike length (**Figures 1a–f**). Increased spikelet density (averaging 3.0 spikelets per cm) was observed on compact spikes in the field; in contrast, decreased spikelet density was also observed as a spelta spike mutation. Wild type spikelet density was 2.3 ± 0.1 spikelets per cm. The mutation frequency of spikelet density was relatively higher than other morphological mutations.

Interestingly, two degenerated spike mutations were obtained (**Figure 1b**). Unlike the lower spikelet, the upper spikelets showed much slower development starting at spikelet emergence and ceased growth completely after heading. This variation showed a mutated phenotype without segregation in the M_3_ plants, indicating that they are regulated by a single recessive gene or genes.

#### Leaf Morphology

Leaf size and chlorophyll content variations (**Figures 1g–n**) were screened from the seedling stage until maturity. Degenerated leaf mutations (**Figures 1i,j**) were observed in the elongation stage, where the flag leaves were completely degenerated. A hypersensitive reaction-like mutation appeared throughout the entire growth period (**Figure 1k** second from right and **Figure 1n**). This mutation was hereditable and the necrotic size varied among different mutagenized wheat lines. The chlorotic leaf mutant showed a gradient leaf color from petiole to apex from green to yellow (**Figure 1k** right and **Figure 1m**).

#### Plant Architecture

The wild type *Jing 411* showed semi-erect growth before the elongation stage, where its height was about 90 cm. Plant architecture variation was observed in the 1.0 and 1.5% mutagenized population in the plant height, growth habit, tiller number, and plant wax phenotypes. Plant height variation was classified into three groups, including two taller lines (>95 cm), six semi-dwarfing (50–80 cm, **Figure 1q**) and two dwarfing (<50 cm) mutants. Three mutant lines showed prostrate phenotypes before the elongation stage (**Figure 1p**). Greater or fewer tiller amounts compared to the wild type were also obtained including a single tiller mutant (**Figures 1r,s**).

#### Seed Morphology

Mutations on grain color (**Figure 1t**), grain size and weight (**Figure 1u**), and grain shape (**Figure 1v**), were observed. The mutation frequency in grain morphology in each EMS treatment was higher than mutation frequency observed in any other traits (**Figure 2**). Grain color of the wild type is white. A single red color mutant was identified in the 1.0% mutagenized population.

### Allelic Variation in the *TaAGP.L-B1* Gene

Two primers specific to the gene *TaAGP.L-B1* were designed and used for allelic variation screening in the M_2_ population (*n* = 1218) treated with 0.5, 1.0, and 1.5% EMS (*n* = 312, 489, and 417, respectively), and 18 mutations were identified in 1.0 and 1.5% subpopulation with 72% transitions (C > T, G > A) and 22% transversions (C > A, T > C, A > C) (**Table 1** and Supplementary Table S2), mutation density was 1/35 and 1/104 Kb respectively, none was screened in 0.5% subpopulation. Four missense mutations and one splice mutation were identified (**Table 1**). One of the missense mutations in line E3-1-3 resulted in a change from proline to leucine in the 378th amino acid. The amino acid site is located in a conserved region in wheat, maize, rice, and potato (**Supplementary Figure S3**). The PSSM and SIFT values also predicted a mutation in line E3-1-3 that might affect gene function. The other three missense mutations in lines E049-4, E422, and E051-6 had PSSM and SIFT values that predicted no significant alteration on function. Furthering analysis on gene expression and starch content were carried out to verify allelic effects.

**Table 1:** Missense, synonymous, and splice mutations identified in gene *TaAGP.L-B1*.

EMS concentration (%)	Line	Nucleotide change	Amino acid change	Zygosity	PSSM value	SIFT score
1.0	E049-4	G1748A	S261N	Homo	2	0.37
1.0	E2-1-43	C1938A	A290=	Homo		
1.5	E3-1-3	C2531T	P378L	Homo	11.2	0.34
1.0	E038-3	G2677A	Splice Junction	Homo		
1.5	E422	G2889A	M436I	Homo	8.7	0.22
1.0	E2-1-32	G2973A	E464=	Homo		
1.0	E051-6	G3116A	G480R	Homo		

*Nucleotide number begins at the start codon ATG*.

### Expression Effects in Developing Seeds from the Novel *TaAGP.L-B1* Alleles

**FIGURE 3:**
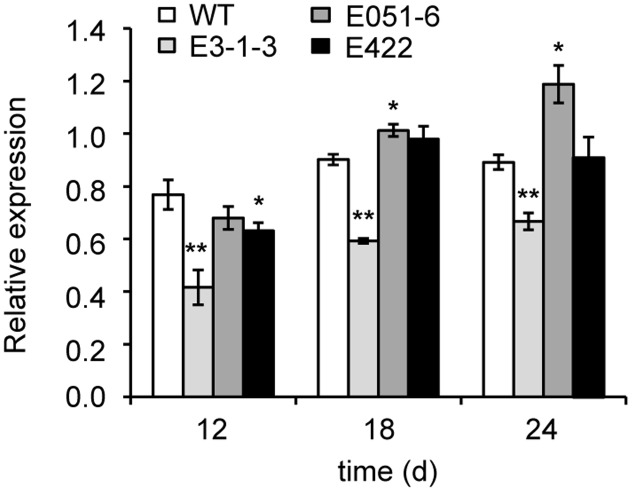
Expression change of gene *TaAGP.L-B1* in developing seeds. Fold change is normalized using the *actin* gene (GenBank accession no: AAW78915) as a control. Three mutated lines in the *TaAGP.L-B1* gene were compared to the wild type wheat *Jing411*. WT, wild type; ^∗^, ^∗∗^ significantly different from WT at the *P* = 0.05, 0.01 level, respectively, based on the Student’s *t*-test.

The *TaAGP.L-B1* expression patterns in developing seeds with missense mutations (lines E051-6, E422, and E3-1-3) were analyzed. Expression levels of the *TaAGP.L-B1* gene in mutant E3-1-3 were significantly reduced (*P* < 0.01) compared to the wild type at 12, 18, and 24 DAF (**Figure 3**). The mutant line E051-6 showed significant change in gene expression at 18 DAF and 24 DAF compared to the wild type (*P* < 0.05). The E422 mutant line only showed a significant change in gene expression compared to the wild type at 12 DAF (*P* < 0.05).

### Effects of Novel Allele on Starch Content

Starch content in the wild type and mutant M_3_ and M_4_ seeds were measured. In the mutated line E3-1-3, starch content showed a significant decrease compared to the wild type, while no significant changes were observed in the other two mutants (**Table 2**).

**Table 2:** Differences in starch content of mature seeds.

Line	Starch content (%)
WT	61.96 ± 3.59 A
E051-6	61.59 ± 0.34 A
E422	62.06 ± 2.36 A
E3-1-3	55.43 ± 3.42 C

*Values are described as means ± standard deviation; labeled with different capital letters denote significant differences at the *P* < 0.01 level based on the Student’s *t*-test*.

## Discussion

### EMS Treatment and Mutation Frequency

Damaging effects in the M_1_ population are closely related with chemical concentration, treatment time, temperature, seed physiological state, and genotype. Typical EMS treatments are carried out using dry seeds directly soaked in solutions at room temperature for 16–18 h in concentration ranging from 0.6 to 1.2% (Slade et al., 2005; Rakszegi et al., 2010; Chen et al., 2012). As germinated seed is more sensitive than dry seed (Lin et al., 1991), we pre-treated our seeds in tap water to achieve germination and only treated seeds for 4 h. Treated seedlings were half the size of the untreated wild type at the 1.0% EMS treatment (**Supplementary Figure S1c**), allowing treatment time to be remarkably shortened. Temperature is an important element in our treatment process to keep seeds in an appropriate physiological state. Here, we treated seeds at 20°C for 4 h with a 10 h pre-treatment of germinating. Higher temperatures would cause physiological and biochemical reactions to occur faster, in turn leading to severe damage effects. To avoid this, treatment times should be shortened. On the contrary, low temperatures result in minor damage effects, allowing for treatment times to be longer.

Gene mutation density in multi-ploid species is higher than in diploid species (Parry et al., 2009). It has been shown that high phenotypic mutation frequency can also be generated in a mutagenized durum wheat population (Bovina et al., 2014). In the present study, the single nucleotide mutation density in 1.0% subpopulation was similar with those in other hexaploid wheat (Slade et al., 2005; Uauy et al., 2009; Chen et al., 2012); while in 1.5% subpopulation was relatively lower. But phenotypic mutation frequencies were different, it ranged from 4 to 19% and increased linearly with increased EMS concentration. The relationship of nucleotide mutation density and phenotypic mutation frequency in mutagenized population need further analysis based on more target genes and larger population. However, mutation frequency varied among phenotypes and EMS treatments (Supplementary Table S1). As reported, EMS treatment resulted in more G/C to A/T transitions than other mutation types in rice, maize, soybean and wheat (Till et al., 2004, 2007; Slade et al., 2005; Cooper et al., 2008; Chen et al., 2012). In our population, 72% mutation type was G/C to A/T transitions, it reconfirmed that EMS induced changes are transitions especially G/C to A/T.

### Phenotypic Mutation Type Is Closely Related with the Genotype of Wild Type

Cultivars arise from long-term evolution and artificial selection and their gene regulatory networks have been optimized during this process. However, mutation induction leads to sequence changes in one or more genes and can break the balance of this network. The majority of mutations result in negative effects on agronomic traits. The *Jing411* winter wheat wild type used in this study is an elite cultivar with high yield potential. Its TGW is about 50 g, showing high quality wheat. In many mutants, the TGW was lower. Our result showed that the maximum mutation frequency was observed in seed morphology, followed by spike morphology.

Numerous phenotypic mutations have been identified in previous reports, but the specific mutated traits in each wild type cultivar were different (Rakszegi et al., 2010; Chen et al., 2012; Bovina et al., 2014; Dhaliwal et al., 2015). These mutant lines, including ours, could be used for further novel gene discovery. More plant height mutations have been observed in mutagenized populations of *Indian* and *Jinmai 47* and this could be a result of both wild type heights being greater than other wheat cultivars (Chen et al., 2012; Dhaliwal et al., 2015). Here, we did not see much phenotypic variation in plant height in the mutagenized lines. Two of the semi-dwarfing mutants from our *Jing 411* mutated resource carried the semi-dwarfing genes *Rht-D1b*, which is the mutation of the allele *Rht-D1a* (Xiong et al., in press). We deduced that mutation type and frequency could be closely related to the genes that are available in the wild type. More mutant resources derived from different wild type cultivars with far genetic distances could produce different phenotypic and genetic mutations that could ultimately be used to increase cultivar quality.

### Non-lethal Allele in Large Subunit Gene AGPase

The large subunit gene of the AGPase enzyme is expressed during grain development (Smidansky et al., 2007; Tang et al., 2016), and the overexpression of this gene results in enhanced AGP activity and starch content (Li et al., 2011; Tuncel and Okita, 2013). The altered amino acid residue in mutant E3-1-3 was located in a conserved region shared with potato and rice. The specific residue interacts with amino acids of the small subunit (Tuncel et al., 2008; Dawar et al., 2013). We did not observe any other mutations in this region. In order to validate the effect of this novel mutated allele, we analyzed the change in AGPase expression at 12, 18, and 24 DAF, as these times are peak expression periods (Thorneycroft et al., 2003; Stamova et al., 2009; Zhang et al., 2016). We found that expression was significantly down-regulated in the mutant E3-1-3, and led to a significant decrease in starch content compared to the wild type. This is consistent with transgenic results (Tuncel and Okita, 2013). Our results confirmed that the mutant E3-1-3 carried a novel allele, which was non-lethal and might related with functional variation of AGPase large subunit gene, it could be used for further analysis of the AGPase large subunit gene effect on wheat growth and development.

### Non-lethal Alleles and Functional Analysis on Multi-ploid Species

Mutagenesis generates mutations randomly and usually produces more than one gene/allele mutation in an individual mutant line. These multi-genic mutations might demonstrate an effect on a phenotypic trait. To reduce mutation background, backcrossing is one of the most commonly used strategies (Konik-Rose et al., 2007; Sestili et al., 2015; Mizuno et al., 2016; Acevedo-Garcia et al., 2017). However, it may take several years for hexaploid wheat crossings and the development of triple mutant lines. Utilization of multiple independent mutants is another alternative strategy (Krasileva et al., 2017). In the present study, we compared expression and phenotypic results of line E3-1-3 with the other two mutant lines E051-6 and E422. The PSSM value of lines E051-6 and E422 were less than 10 and were predicated to have no severe effect. The gene expression patterns and starch contents in these two lines varied slightly, indicating that background mutations did not affect AGPase large subunit gene function. The starch content variation in the mutant E3-1-3 resulted from a novel allele in *TaAGP.L-B1*. Similarly, mutations on gene *TaSSIVb-D* have been identified in a mutagenized population, and by directly using two mutant lines, the gene function of *TaSSIVb-D* on transient starch synthesis in wheat leaves was elucidated (Guo et al., 2017). This suggests that using multiple individual mutant lines to illustrate gene characterization in multi-ploid species is an effective strategy for reverse genetics at least in major genes. Contribution of each homoeologous loci might be different (Yu et al., 2012; Yang et al., 2014), expression variations and phenotypic changes caused by gene mutation would be different either, those with higher genetic effects would be much easier to be analyzed. Genetic effects and their difference of the three homoeologous of *TaAGP.L* need to be verified by further experiments.

## Conclusion

A new genetic resource with phenotypic variation and non-lethal alleles was developed using a hexaploid winter wheat cultivar as the wild type. This resource could be used for wheat forward and reverse genetic research to improve wheat agronomic traits.

## Author Contributions

HG wrote the manuscript, HG, ZY, XL, and YL carried out allele identification and function analysis; HG, YX, LZ, JG, and SZ developed the mutated population; LL conceived the original research, supervised data generation and analyses and wrote the manuscript. All authors read and approved the final manuscript.

## Conflict of Interest Statement

The authors declare that the research was conducted in the absence of any commercial or financial relationships that could be construed as a potential conflict of interest.

## Footnotes

1 http://blocks.fhcrc.org/proweb/parsesnp/

2 http://sift.bii.a-star.edu.sg/

## Supplementary Material

FIGURE S1 Seeds used in EMS treatment and seedlings post-EMS treatment. **(a)** Dry seeds; **(b)** germinated seeds for EMS treatment; **(c)** seedlings after EMS treatment.

FIGURE S2 Diagram of the gene *TaAGP.L-B1* structure. The black rectangle represents the genomic sequence of the gene; orange arrowheads and rectangles indicate exons and their locations; blue rectangles represent 2 sets of specific primers.

FIGURE S3 Alignment of the amino acid sequence of the AGPase large subunit. Black rectangles show wheat line point mutations in amino acid sites resulting from EMS-treatment. Sequences were aligned using ClustalX, Accession numbers of potato, maize, and rice are Q00081.1, P55241.1, and ACJ71342.1 respectively.
