# Supplementary figures and images for: Development of a High-Efficient Mutation Resource with Phenotypic Variation in Hexaploid Winter Wheat and Identification of Novel Alleles in the TaAGP.L-B1 Gene
Source: Front Plant Sci. 2017 Aug 10;8:1404. doi: 10.3389/fpls.2017.01404 (PMC5554398; doi:10.3389/fpls.2017.01404)

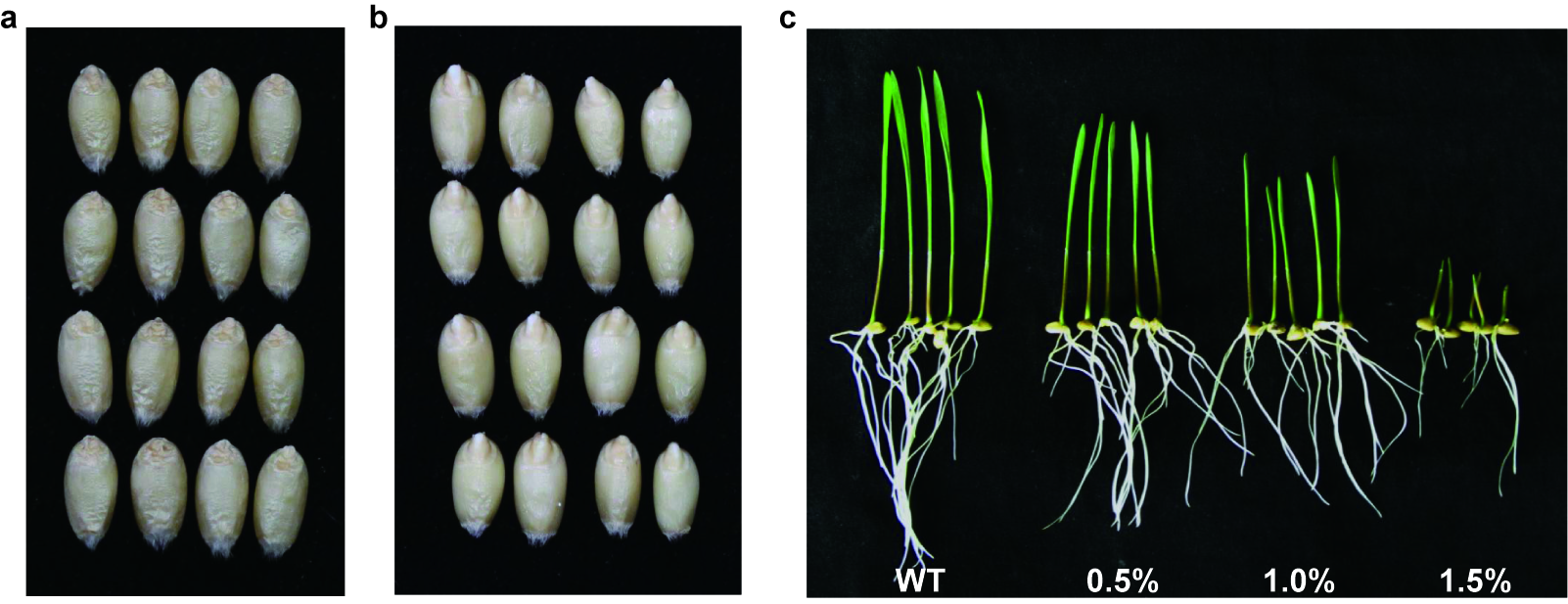

Supplement: FIGURE S1 — Seeds used in EMS treatment and seedlings post-EMS treatment. (a) Dry seeds; (b) germinated seeds for EMS treatment; (c) seedlings after EMS treatment. [file Image_1.TIF]

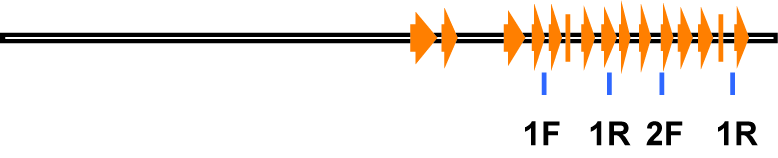

Supplement: FIGURE S2 — Diagram of the gene TaAGP.L-B1 structure. The black rectangle represents the genomic sequence of the gene; orange arrowheads and rectangles indicate exons and their locations; blue rectangles represent 2 sets of specific primers. [file Image_2.TIF]

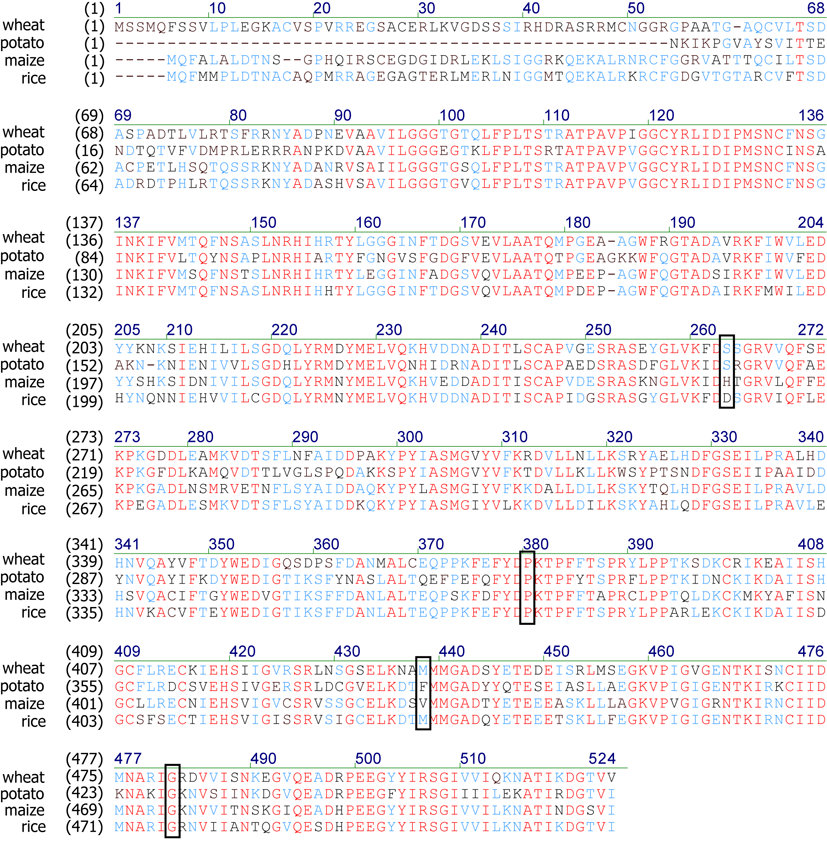

Supplement: FIGURE S3 — Alignment of the amino acid sequence of the AGPase large subunit. Black rectangles show wheat line point mutations in amino acid sites resulting from EMS-treatment. Sequences were aligned using ClustalX, Accession numbers of potato, maize, and rice are Q00081.1, P55241.1, and ACJ71342.1 respectively. [file Image_3.TIF]
