# Supplementary material for: Development of a High-Efficient Mutation Resource with Phenotypic Variation in Hexaploid Winter Wheat and Identification of Novel Alleles in the TaAGP.L-B1 Gene
Source: Front Plant Sci. 2017 Aug 10;8:1404. doi: 10.3389/fpls.2017.01404 (PMC5554398; doi:10.3389/fpls.2017.01404)
Supplement: Supplementary file 4 [file Table_1.docx]

**Table S1.** Mutated phenotype and mutation frequency in mutagenized populations

| Category | Phenotype | | | 0.5 % 4 h | | | | | |  | 1.0 % 4 h | | | | | | |  | 1.5 % 4 h | | | | |
| --- | --- | --- | --- | --- | --- | --- | --- | --- | --- | --- | --- | --- | --- | --- | --- | --- | --- | --- | --- | --- | --- | --- | --- |
|  |  |  |  | No. | Mutation frequency % | | | | |  | No. | Mutation frequency % | | | | | |  | No. | Mutation frequency % | | | |
| Heading data | Earlier heading | | 5 | 1.60 | | | |  | 3 | | 0.61 | | | |  | 2 | | | 0.48 | | | |  |
|  | Later heading | | - | - | | | |  | 4 | | 0.82 | | | |  | 6 | | | 1.44 | | | |  |
| Spike morphology | Compact spike | | 2 | 0.64 | | | |  | 8 | | 1.64 | | | |  | 13 | | | 3.12 | | | |  |
|  | Square spike | | - | - | | | |  | 1 | | 0.20 | | | |  | - | | | - | | | |  |
|  | Spelta spike | | - | - | | | |  | 4 | | 0.82 | | | |  | 4 | | | 0.96 | | | |  |
|  | Degenerated spike | | - | - | | | |  | - | | - | | | |  | 2 | | | 0.48 | | | |  |
|  | awnless | | - | - | | | |  | 1 | | 0.20 | | | |  | 1 | | | 0.24 | | | |  |
|  | Male sterility | | - | - | | | |  | 2 | | 0.41 | | | |  | 2 | | | 0.48 | | | |  |
| Leaf morphology | Hypersensitive reaction like | | - | - | | | |  | 1 | | 0.20 | | | |  | 2 | | | 0.48 | | | |  |
|  | Albino | | - | - | | | |  | 1 | | 0.20 | | | |  | - | | | - | | | |  |
|  | Yellow-striped leaf | | 1 | 0.32 | | | |  | - | | - | | | |  | - | | | - | | | |  |
|  | Yellowing leaf | | |  |  | | | | |  |  |  | | | | | |  | 1 | 0.24 | | | |
|  | Narrow leaf | - | - | | |  | 1 | | 0.20 | | | |  | | - | | - | | | | |  |  |
|  | Curly leaf | | | - | - | | | | |  | - | - | | | | | |  | 4 | 0.72 | | | |
|  | Degenerated leaf | | | - | - | | | | |  | - | - | | | | | |  | 2 | 0.48 | | | |
| Plant architecture | Prostrate-type |  |  | | |  | 1 | | 0.20 | | | |  | 2 | | 0.48 | | | | |  |  |  |
|  | Dwarfing and semi-dwarfing |  |  | | |  | 3 | | 0.61 | | | |  | 5 | | 1.20 | | | | |  |  |  |
|  | Tall plant | - | - | | |  | 1 | | 0.20 | | | |  | 1 | | 0.24 | | | | |  |  |  |
|  | More tiller | - | - | | |  | 1 | | 0.20 | | | |  | - | | - | | | | |  |  |  |
|  | few tiller | - | - | | |  | 4 | | 0.82 | | | |  | - | | - | | | | |  |  |  |
|  | Wax-less plant | - | - | | |  | - | | - | | | |  | 1 | | 0.24 | | | | |  |  |  |
| Seed morphology | Red grain | - | - | | |  | - | | - | | | |  | 1 | | 0.24 | | | | |  |  |  |
|  | Thousand grain weight | 2 | 0.64 | | |  | 15 | | 3.07 | | | |  | 17 | | 4.08 | | | | |  |  |  |
|  | Wrinkle grain | 3 | 0.96 | | |  | 6 | | 1.23 | | | |  | 10 | | 2.40 | | | | |  |  |  |
|  | Round grain | - | - | | |  | 1 | | 0.20 | | | |  | 5 | | 1.20 | | | | |  |  |  |
|  | Long grain | - | - | | |  | 2 | | 0.41 | | | |  | - | | - | | | | |  |  |  |
|  | Thin grain | - | - | | |  | 1 | | 0.20 | | | |  | - | | - | | | | |  |  |  |
|  | Small grain | - | - | | |  | 1 | | 0.20 | | | |  | - | | - | | | | |  |  |  |
| Mutation frequency % | | 13 | 4.15 | | |  | 62 | | 12.68 | | | |  | 80 | | 19.18 | | | | |  |  |  |

**Table S2. Nucleotide changes identified in the intron region of gene *TaAGP.L-B1***

| EMS Concentration | Line | Nucleotide Change | Mutation Type | Zygosity |
| --- | --- | --- | --- | --- |
| 1.0% | E2-2-235 | -1867T^*^ | Intron | Homo |
| 1.0% | E2-2-171 | G1891A | Intron | Homo |
| 1.0% | E2-2-21 | G1897R | Intron | Hetero |
| 1.0% | E2-2-434 | C2610T | Intron | Homo |
| 1.0% | E2-2-219 | G2613A | Intron | Homo |
| 1.0% | E040-15 | G2787A | Intron | Homo |
| 1.0% | E2-1-43 | T2791C | Intron | Homo |
| 1.5% | E313 | G2854R | Intron | Hetero |
| 1.5% | E370 | G3149A | Intron | Homo |
| 1.0% | E038-3 | A3213C | Intron | Homo |
| 1.0% | E2-2-62 | A3213C | Intron | Homo |

^*^: insertion mutation
